# Deep Learning-Based Classification of Macrofungi: Comparative Analysis of Advanced Models for Accurate Fungi Identification

**DOI:** 10.3390/s24227189

**Published:** 2024-11-09

**Authors:** Sifa Ozsari, Eda Kumru, Fatih Ekinci, Ilgaz Akata, Mehmet Serdar Guzel, Koray Acici, Eray Ozcan, Tunc Asuroglu

**Affiliations:** 1 Department of Computer Engineering, Faculty of Engineering, Ankara University, Ankara 06830, Türkiye; ozsaris@ankara.edu.tr (S.O.); mguzel@ankara.edu.tr (M.S.G.); eozcan@teus.com.tr (E.O.); 2 Graduate School of Natural and Applied Sciences, Ankara University, Ankara 06830, Türkiye; edakumru98@gmail.com; 3 Department of Medical Physics, Institute of Nuclear Sciences, Ankara University, Ankara 06100, Türkiye; fatihekinci@ankara.edu.tr; 4 Department of Biology, Faculty of Science, Ankara University, Ankara 06100, Türkiye; akata@science.ankara.edu.tr; 5 Department of Artificial Intelligence and Data Engineering, Faculty of Engineering, Ankara University, Ankara 06830, Türkiye; kacici@ankara.edu.tr; 6 Faculty of Medicine and Health Technology, Tampere University, 33720 Tampere, Finland; 7 VTT Technical Research Centre of Finland, 33101 Tampere, Finland

**Keywords:** macrofungi classification, deep learning, DenseNet121, fungi identification, machine learning models

## Abstract

This study focuses on the classification of six different macrofungi species using advanced deep learning techniques. Fungi species, such as *Amanita pantherina*, *Boletus edulis*, *Cantharellus cibarius*, *Lactarius deliciosus*, *Pleurotus ostreatus* and *Tricholoma terreum* were chosen based on their ecological importance and distinct morphological characteristics. The research employed 5 different machine learning techniques and 12 deep learning models, including DenseNet121, MobileNetV2, ConvNeXt, EfficientNet, and swin transformers, to evaluate their performance in identifying fungi from images. The DenseNet121 model demonstrated the highest accuracy (92%) and AUC score (95%), making it the most effective in distinguishing between species. The study also revealed that transformer-based models, particularly the swin transformer, were less effective, suggesting room for improvement in their application to this task. Further advancements in macrofungi classification could be achieved by expanding datasets, incorporating additional data types such as biochemical, electron microscopy, and RNA/DNA sequences, and using ensemble methods to enhance model performance. The findings contribute valuable insights into both the use of deep learning for biodiversity research and the ecological conservation of macrofungi species.

## 1. Introduction

Macrofungi, a group of fungi producing prominent fruiting bodies above or below the ground, represent an immense diversity within the species Basidiomycota and Ascomycota under the kingdom Fungi. These fruiting bodies, commonly known as mushrooms, are easily identifiable in natural environments and are vital components of forest ecosystems [1]. Macrofungi sustain ecosystem functionality by contributing to nutrient cycling, soil formation, and complex biotic interactions with other organisms [2]. To date, over 56,000 species of macrofungi have been documented worldwide, highlighting their remarkable biodiversity and ecological significance. Notably, approximately 1000 species are considered toxic, while more than 2000 species are edible, providing nutritional value and supporting food security for both humans and forest fauna [2,3,4,5].

Among the diverse macrofungi, many species exhibit unique ecological relationships and have significant cultural, nutritional, or toxicological relevance. For instance, *Amanita pantherina*, also known as the panther cap, is associated with coniferous and deciduous woodlands and has a storied history in mystical practices due to its psychoactive components, ibotenic acid and muscimol [6,7]. In contrast, *Boletus edulis*, or porcini, is one of the most popular wild edible mushrooms, valued for its rich nutritional content, including proteins, vitamins, and essential minerals [8]. Similarly, *Cantharellus cibarius*, the chanterelle, is cherished not only for its flavor and culinary value but also for its significant levels of bioavailable vitamin D2, crucial for human health [9].

Other notable macrofungi include *Lactarius deliciosus*, or the delicious milk cap, which plays an essential ecological role as an ectomycorrhizal partner in pine forests, promoting their growth and health [10]. Likewise, *Pleurotus ostreatus*, commonly known as the oyster mushroom, is not only a commercially important edible species but also contributes to environmental sustainability through the bioconversion of agricultural waste [11,12]. In contrast, *Tricholoma terreum*, previously considered a desirable edible species, has been re-evaluated due to potential health risks linked to rhabdomyolysis, underscoring the complex interplay between macrofungi and human health [13].

The advancement of machine learning (ML) and computer vision technologies is revolutionizing macrofungi identification and classification, offering significant advantages over traditional taxonomic methods. Conventional identification requires extensive expertise and can be time-consuming, whereas ML-based systems can rapidly analyze large datasets, discerning even subtle morphological variations with high precision [14,15]. Such technological integration accelerates species identification and enhances accessibility for non-experts through user-friendly mobile applications, thereby reducing misidentification risks and promoting broader public engagement in fungal biodiversity [16]. Furthermore, incorporating environmental data into ML models enhances ecological studies, contributing to the conservation of macrofungal species [17]. The synergy between researchers and citizen scientists has proven instrumental in creating high-quality datasets essential for effective conservation strategies.

The current research aims to leverage deep learning (DL), an advanced subset of artificial intelligence, to classify six distinct macrofungi species, namely *Amanita pantherina*, *Boletus edulis*, *Cantharellus cibarius*, *Lactarius deliciosus*, *Pleurotus ostreatus*, and *Tricholoma terreum*. These species were selected based on their ecological significance and diverse morphological characteristics. The development of a DL model for the rapid and accurate identification of these species will support scientific research and enhance public awareness and engagement, ultimately contributing to the conservation and sustainable use of macrofungal biodiversity.

## 2. Materials and Methods

**Figure 1:**
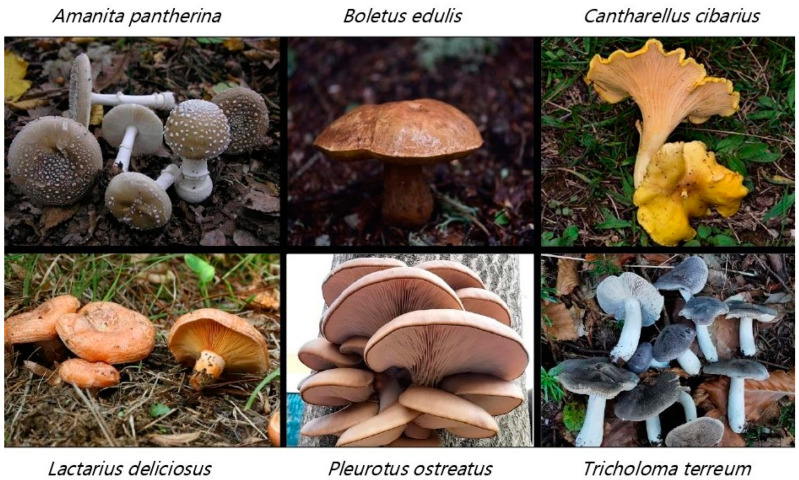
Overview of datasets utilized for training AI algorithms, presented from a macroscopic perspective.

In this study, datasets were generated using naturally captured images, subsequently labeled for the training of artificial intelligence (AI) algorithms (Figure 1). Data deemed unsuitable for training purposes were excluded from the final dataset. The images were then converted into the PNG format, with a resolution of 300 dpi to ensure consistency across the dataset. Given the requirement for a large and suitable dataset for AI algorithm training, additional data were sourced from open access repositories to supplement the natural data. Specifically, open-source data (www.gbif.org, accessed on 8 October 2024) accounted for no more than 25% of the total data for each mushroom species. Detailed information regarding the sources and characteristics of the datasets derived from these open sources is presented in Table 1. The primary objective was to train AI algorithms; thus, the images were not presented in their raw form within the article. Considering the substantial data requirements for effective AI training, it became evident that obtaining a dataset of the required size solely from natural shots would be impractical. Therefore, publicly available datasets were utilized to bridge this gap and meet the necessary data volume for robust AI training.

Deep learning has evolved significantly since its early foundations in the 1940s. The concept of artificial neurons, introduced by Warren McCulloch and Walter Pitts [18], laid the groundwork for neural networks, followed by Frank Rosenblatt’s perceptron in 1957 [19]. However, neural networks faced limitations, particularly with non-linear problems, which led to a decline in interest after criticisms in the late 1960s. The field resurged in the 1980s with the reintroduction of backpropagation by Geoffrey Hinton and colleagues [20], allowing multi-layer networks to be trained more effectively. Despite early progress, deep learning faced computational and data limitations until the 2000s, when breakthroughs like Hinton’s deep belief networks and Alex Krizhevsky’s convolutional neural networks (CNNs) [21] in the 2012 ImageNet competition demonstrated the power of deep architectures. The availability of GPUs and large datasets accelerated these advancements. The late 2010s saw further breakthroughs with the development of generative models like GANs and transformers, which revolutionized fields like image generation and natural language processing. Today, deep learning thrives due to improved algorithms, big data, and computational power, driving innovation across industries from healthcare to robotics.

In this study, 5 different machine learning techniques, logistic regression, Support Vector Machine (SVM), k-Nearest Neighbors (k-NN), decision tree, and random forest algorithms, and 12 different deep learning methods, ConvNeXtBase [22], ConvNeXtSmall [22], ConvNeXtLarge [22], EfficientNetB [23], EfficientNetB3 [23], EfficientNetB7 [23], DenseNet121 [24], InceptionV3 [25], InceptionResNetV2 [26], MobileNetV2 [27], ResNet152 [28], Xception [29], and swin transformers (shifted window transformers) [30] were used for the classification of six different fungi species.

### 2.1. ML

Logistic regression [31] is a linear model used for binary classification tasks. It estimates the probability that a given input belongs to a specific class by applying the logistic function to a linear combination of the input features. The output is a probability score between 0 and 1, which is then used to classify the instance. It works best when the classes are linearly separable and is easy to implement and interpret. However, it may struggle with more complex relationships in the data.

SVM [32] is a powerful classification algorithm that seeks to find the optimal hyperplane that best separates the classes in the feature space. It works by maximizing the margin between data points from different classes. SVM is effective in high-dimensional spaces and with non-linearly separable data when using kernel tricks. Despite its power, SVM can be sensitive to noisy data and may require the careful tuning of parameters (such as the choice of kernel) to achieve optimal performance.

k-NN [33] is a non-parametric, instance-based learning algorithm that classifies a new data point based on the majority class among its “k” nearest neighbors. It does not assume any underlying distribution of the data, making it versatile. However, k-NN can be computationally expensive with large datasets, and it is sensitive to the choice of “k” and the distance metric. It also tends to struggle with noisy data, as each neighbor’s class significantly impacts the result.

Decision tree [34] is a tree-structured model that splits the data into subsets based on feature values, making decisions using a series of if-then conditions. Each internal node represents a feature, each branch represents a decision rule, and each leaf node represents an outcome (class label). Decision trees are highly interpretable but prone to overfitting, especially if the tree grows too deep. They are non-linear models and work well for both classification and regression tasks.

Random forest [35] is an ensemble learning method that builds multiple decision trees during training and merges their predictions to improve accuracy and reduce overfitting. Each tree is built using a random subset of features and data points, which introduces variability and makes the model more robust than a single decision tree. Random forest can handle large datasets with higher dimensionality but may become less interpretable due to the combination of multiple trees.

### 2.2. DL

ConvNeXtBase [22] is a modern convolutional neural network (CNN) architecture designed as an evolution of traditional CNNs, integrating ideas from transformer models while maintaining the efficiency and simplicity of CNNs. Developed by Facebook AI Research, it adopts design principles from the vision transformer (ViT), such as using large kernel sizes and layer normalization, while refining conventional CNN elements. ConvNeXtBase is known for its strong performance in image classification tasks, achieving competitive results with transformers on large-scale datasets like ImageNet, while being more computationally efficient for vision tasks. This model balances high accuracy and efficiency, making it suitable for a variety of visual recognition applications.

ConvNeXtSmall [22] is a variant of the ConvNeXt architecture, designed to offer a more lightweight and efficient model for image classification and other vision tasks. Like ConvNeXtBase, it builds on traditional CNN structures while incorporating innovations inspired by transformer models, such as large kernel sizes and layer normalization. ConvNeXtSmall maintains a balance between computational efficiency and accuracy, making it well-suited for scenarios where lower computational cost is important, but strong performance on tasks like object recognition is still required. It delivers competitive results on benchmarks like ImageNet, offering a good trade-off between speed and performance for real-world applications.

ConvNeXtLarge [22] is a larger and more powerful variant of the ConvNeXt architecture, designed for high-performance image classification and visual recognition tasks. Like its smaller counterparts, ConvNeXtLarge combines the strengths of traditional convolutional neural networks with design elements from vision transformers, such as large kernel sizes, layer normalization, and attention mechanisms. It is more computationally intensive but delivers top-tier performance, particularly on large-scale datasets like ImageNet. ConvNeXtLarge is particularly well-suited for tasks that demand higher accuracy and robustness, often outperforming other models in terms of precision while maintaining the efficiency associated with CNN-based architectures.

EfficientNetB0 [23] is the smallest model in the EfficientNet family of convolutional neural networks, designed to achieve high accuracy while being computationally efficient. Developed by Google, EfficientNetB0 uses a novel compound scaling method that uniformly scales network depth, width, and resolution to optimize performance. This approach allows the model to achieve excellent results on benchmarks like ImageNet with significantly fewer parameters and lower computational costs compared to traditional architectures. EfficientNetB0 is particularly useful in resource-constrained environments where both accuracy and efficiency are essential, making it ideal for mobile and edge applications.

EfficientNetB3 [23] is a larger and more powerful variant within the EfficientNet family, designed to balance accuracy and efficiency using Google’s compound scaling method. Like EfficientNetB0, it scales depth, width, and resolution uniformly, but with more layers and higher resolution inputs, leading to improved performance on tasks such as image classification. EfficientNetB3 achieves better accuracy than smaller models like B0 while still being more computationally efficient compared to other architectures of similar accuracy. It is well-suited for scenarios where higher accuracy is needed but computational resources are somewhat limited, offering strong performance on benchmarks like ImageNet with manageable resource requirements.

EfficientNetB7 [23] is the largest and most powerful model in the EfficientNet family, designed for high-performance tasks where maximizing accuracy is a priority. Like other EfficientNet models, it uses Google’s compound scaling method to effectively scale up the network’s depth, width, and input resolution. EfficientNetB7 offers superior accuracy on image classification benchmarks like ImageNet, often achieving state-of-the-art results. However, due to its larger size and higher computational demands, it requires more resources compared to smaller models like EfficientNetB0 or B3. EfficientNetB7 is best suited for tasks that demand high precision and where computational resources are more readily available.

DenseNet121 [24] is a convolutional neural network model that belongs to the DenseNet (Densely Connected Networks) family, introduced to improve information flow between layers. Unlike traditional architectures where layers are connected sequentially, DenseNet121 connects each layer to every other layer in a feed-forward manner. This dense connectivity encourages feature reuse, reduces the vanishing gradient problem, and improves parameter efficiency, allowing the model to be relatively compact while maintaining high performance. DenseNet121 has 121 layers and is particularly effective for image classification tasks, offering strong results on benchmarks like ImageNet while requiring fewer parameters and less computational power than other deep models, such as ResNet. Its efficient design makes it well-suited for various computer vision applications, especially when computational resources are limited.

InceptionV3 [25] is a deep convolutional neural network architecture that is part of Google’s Inception family, known for its efficient use of computational resources. It builds on the earlier versions of Inception by refining the “Inception module” concept, where multiple convolutional filters of different sizes are applied to the same input in parallel. This allows the network to capture a wider range of spatial features, increasing its ability to recognize complex patterns in images. InceptionV3 introduces additional improvements, such as factorized convolutions (splitting a large convolution into smaller ones), grid reduction techniques, and the use of batch normalization to improve training speed and accuracy. It is highly effective for image classification tasks and has achieved strong results on large-scale datasets like ImageNet, offering a good balance between high accuracy and computational efficiency. InceptionV3 is widely used in real-world applications, such as object detection and image analysis, especially when resources are constrained but high performance is required.

MobileNetV2 [27] is a convolutional neural network architecture designed specifically for efficient performance on mobile and resource-constrained devices. Introduced by Google, it improves on the original MobileNet by using an inverted residual structure and linear bottlenecks, which help to reduce computational cost while maintaining accuracy. The key innovation in MobileNetV2 is the “inverted residual block”, which expands the input channels first, applies depthwise separable convolutions, and then compresses the output back down, allowing for more efficient processing. MobileNetV2 is highly efficient in terms of both speed and memory usage, making it ideal for applications like real-time image recognition on mobile devices or edge computing. Despite its lightweight nature, it performs well on benchmarks like ImageNet, offering a good trade-off between performance and efficiency for mobile AI tasks.

ResNet152 [28] is part of the ResNet family, which introduced the concept of residual connections to address the vanishing gradient problem that commonly occurs in very deep networks. These connections enable the network to “skip” layers, allowing the model to learn residual mappings instead of direct mappings, which improves training efficiency and accuracy as depth increases. ResNet152, with 152 layers, is one of the deeper variants, utilizing bottleneck blocks to reduce the computational cost while preserving model capacity. This design allows ResNet152 to achieve high accuracy on challenging tasks, such as image classification on ImageNet, while remaining computationally efficient compared to earlier deep networks. Its robustness and scalability have made it a popular backbone for various computer vision applications, including object detection and image segmentation.

InceptionResNetV2 [26] is a hybrid convolutional neural network architecture that combines the strengths of the Inception and Residual networks. This model builds on the concepts introduced in both the InceptionV3 and ResNet architectures, aiming to enhance performance on image classification tasks while maintaining computational efficiency.

Xception [29] is a deep convolutional neural network architecture that builds upon the ideas of depthwise separable convolutions, which were popularized by MobileNets. Introduced by François Chollet, Xception stands for “Extreme Inception” and is designed to enhance model efficiency and performance by separating the spatial and channel-wise convolutions. The architecture consists of a series of depthwise separable convolutions followed by pointwise convolutions, allowing the model to learn rich feature representations while significantly reducing the number of parameters compared to traditional convolutional layers. Xception has achieved state-of-the-art results on image classification tasks, particularly on benchmarks like ImageNet. Its design emphasizes efficient computation and parameter utilization, making it well-suited for both high-performance applications and deployment in resource-constrained environments. This architecture has influenced subsequent models and is widely used in various computer vision applications, including image classification, object detection, and transfer learning.

Swin transformers [36] are a novel vision transformer architecture developed by Microsoft Research, designed to enhance performance on a variety of computer vision tasks while maintaining computational efficiency. This architecture utilizes a hierarchical structure that processes images at multiple scales, making it particularly effective for tasks such as image classification, object detection, and semantic segmentation. A key feature of swin transformers is their use of shifted windows, which divides the input image into non-overlapping windows and performs self-attention within these windows. By shifting the windows at each layer, the model captures cross-window interactions, allowing it to learn relationships between different parts of the image more effectively. Additionally, the hierarchical representation builds feature maps of varying resolutions, capturing both fine and coarse details, while windowed self-attention reduces the computational burden typically associated with global self-attention mechanisms. Swin transformers have achieved state-of-the-art results on benchmark datasets like ImageNet, COCO, and ADE20K, demonstrating their effectiveness across various computer vision applications and representing a significant advancement in applying transformer architectures to vision tasks.

The all-selected DL models each offer unique strengths that make them valuable for a diverse range of tasks. ConvNeXt models (Base, Small, Large) represent a modernized version of traditional CNNs, combining the simplicity of convolutions with updates inspired by ViTs, making them efficient and scalable. EfficientNet (B0, B3, B7) uses a specialized scaling method to balance accuracy and computational efficiency, offering excellent performance with relatively low resource demands. DenseNet121 optimizes feature reuse by connecting each layer to all previous layers, leading to efficient learning with fewer parameters. InceptionV3 and InceptionResNetV2 are designed to capture multi-scale features, with the latter combining Inception’s multi-scale processing and ResNet’s residual connections for improved gradient flow and deeper model training. MobileNetV2 focuses on lightweight architecture, making it ideal for limited computational resources. ResNet152 is a deep network that uses residual connections to ensure stable training and strong performance, even with considerable depth. Xception builds on Inception by replacing standard convolutions with depthwise separable convolutions, offering both efficiency and high accuracy. Finally, swin transformers introduce a transformer-based architecture that efficiently processes images using hierarchical patching, making it well-suited for capturing long-range dependencies in vision tasks. Together, these models provide a broad spectrum of approaches, covering traditional CNNs, efficient neural network designs, and modern transformer-based architectures. Also, they have been widely adopted and validated on popular benchmark datasets, such as ImageNet.

### 2.3. Metrics

The models, ConvNeXtBase, ConvNeXtSmall, ConvNeXtLarge, EfficientNetB0, EfficientNetB3, EfficientNetB7, DenseNet121, InceptionV3, InceptionResNetV2, MobileNetV2, ResNet152, and Xception, were fine-tuned using consistent parameter settings. The Adaptive Moment Estimation (ADAM) optimizer [37] was employed with a learning rate of 0.0001, and the models were trained for 20 iterations.

All experiments were conducted on Google Colaboratory, a cloud-based platform that allows users to write and execute Python code for free, widely used by data scientists, machine learning practitioners, and researchers. To ensure balanced training, equal numbers of data samples were used for each macrofungi species during both training and testing. Given the limited number of available images, 4-fold cross-validation was implemented. This widely used technique is particularly beneficial when working with small datasets, as it divides the data into k equal-sized subsets (folds). The model is trained and validated across k iterations, with a different fold used for validation and the remaining k-1 folds used for training in each round. At each iteration, the model is trained on most of the data and tested on the reserved fold, providing insight into its performance. This process continues until every fold has been used once for validation. Afterward, the results from all iterations are averaged to give a more reliable estimate of the model’s generalization performance. Importantly, the training and testing datasets were kept completely separate, ensuring that no images used for training were included in the testing set.

The effectiveness of all the methods applied was evaluated based on several key performance metrics, including accuracy, which measures the overall correctness of the predictions, precision, which assesses the proportion of correctly identified positive instances out of all predicted positives, and recall, which evaluates the model’s ability to identify actual positive cases. Additionally, the F1-score was used to provide a harmonic mean between recall and precision, giving a balanced measure of the model’s performance, especially on imbalanced datasets. Lastly, the AUC (Area Under the ROC Curve) was included to assess the model’s capability to distinguish between positive and negative classes across varying decision thresholds, with a higher AUC indicating better discriminatory power. The equations for accuracy, precision, recall and F1-score metrics are presented in Equation (1), Equation (2), Equation (3), and Equation (4), respectively.

(1) $$Accuracy=\frac{TP+TN}{TP+TN+FP+FN}$$

(2) $$Precision=\frac{TP}{TN+FP}$$

(3) $$Recall=\frac{TP}{TP+FN}$$

(4) $$F1-score=\frac{2\times (Precision\times Recall)}{Precision+Recall}$$

In Equations (1)–(3), the terms TP, FP, TN, and FN are defined as follows [38]:

- TP (true positives) corresponds to instances where a diseased condition is accurately predicted.
- FP (false positives) occurs when a healthy sample is incorrectly identified as diseased.
- TN (true negatives) refers to cases where healthy samples are correctly classified.
- FN (false negatives) pertains to instances where a diseased condition is wrongly classified as healthy.

Additionally, the Grad-CAM (Gradient-weighted Class Activation Mapping) [39] technique was employed to analyze the specific areas of the images that the models prioritized during the inference process. Neural networks are composed of multiple interconnected layers with numerous parameters that are adjusted during training to process and interpret input data. However, understanding how the models generate outputs from the given inputs can be challenging, often leading to a lack of transparency and reduced confidence in their predictions. Grad-CAM is a powerful visualization method designed to interpret the decision-making processes of convolutional neural networks. It is particularly useful in image classification tasks, as it highlights the regions of an image that are most influential in the model’s prediction. In Grad-CAM visualizations, red regions represent the areas where the model is most focused, meaning that the network uses these regions to form its prediction. Conversely, blue regions signify areas of lesser importance, indicating that the model pays minimal attention to these parts when making its decision. This technique enhances the interpretability of CNNs, offering insights into how the model processes visual data and providing a clearer understanding of its decision-making process.

## 3. Results

This section outlines the experiments carried out and the results obtained from the study.

The ML models were carefully configured with parameters to provide a balance between accuracy, robustness, and computational efficiency. The parameter settings for these models were set as follows:

For logistic regression:

- Solver = liblinear
- Maximum iteration = 1000
- Regularization parameter C = 1.0

For SVM:

- Kernel = Radial Basis Function (RBF)
- Regularization parameter C = 1.0
- Probability = true

For k-NN:

- N neighbors = 5
- weights = distance
- metric = Minkowski with *p* = 2

For decision tree:

- maximum depth = 10
- minimum samples split = 10
- minimum samples leaf = 5

For random forest:

- N estimators = 100
- Maximum depth = 10
- Minimum samples split = 10
- Minimum samples leaf = 5
- Maximum features = sqrt

When Table 2 is examined, logistic regression and SVM emerged as the best-performing models, both achieving an accuracy of 0.60, with balanced precision and recall scores of 0.60. Their F1-scores were slightly lower, at 0.58 and 0.56, respectively, reflecting small trade-offs between precision and recall. Both models showed good discriminatory power, with AUC values of 0.70 for logistic regression and 0.71 for SVM. On the other hand, k-NN performed poorly across all metrics, with an accuracy of only 0.45, a low F1-score of 0.40, and an AUC of 0.50, indicating no better performance than random guessing. The decision tree model also showed weak results, with an accuracy of 0.48 and a similar F1-score of 0.48, though its AUC was slightly higher at 0.53. Random forest performed moderately, with an accuracy of 0.57 and an AUC of 0.60, showing better performance than decision tree but still falling short of logistic regression and SVM. Overall, logistic regression and SVM offered the best trade-off between precision and recall, as well as the strongest ability to distinguish between classes, while k-NN and decision tree struggled with lower accuracy and AUC scores. The overall results indicate that traditional methods struggle to achieve high accuracy or robust class separation in this context. Therefore, they would generally be considered moderate-to-poor performers for this task; more advanced methods, like deep learning, were applied to the problem.

In DL algorithms for data preprocessing, all images were resized to a uniform resolution to fit the input requirements of the selected models, ensuring consistency across the dataset. Images were also normalized by scaling pixel values to the [0, 1] range, which helps improve model convergence by ensuring that input features have similar scales. Additionally, the dataset was checked for any corrupted or mislabeled images, and appropriate corrections were made.

Regarding data augmentation, we employed horizontal and vertical flipping, as well as random rotations. These techniques were chosen to introduce variability in the orientation of the fungi images, helping the models learn to recognize fungi from different angles and perspectives. By augmenting the data in this way, we aimed to improve the model’s robustness and generalization capabilities, ensuring it can handle variations in the positioning of fungi in real-world settings. These straightforward augmentation methods were applied during training to prevent overfitting and improve model performance on unseen data.

Following the data augmentation process, a series of experiments were conducted to assess the effectiveness of transfer learning-based models, namely ConvNeXtBase, ConvNeXtSmall, ConvNeXtLarge, EfficientNetB0, EfficientNetB3, EfficientNetB7, DenseNet121, InceptionV3, InceptionResNetV2, MobileNetV2, ResNet152, Xception, and swin transformers, in predicting six different fungi species automatically.

**Figure 2:**
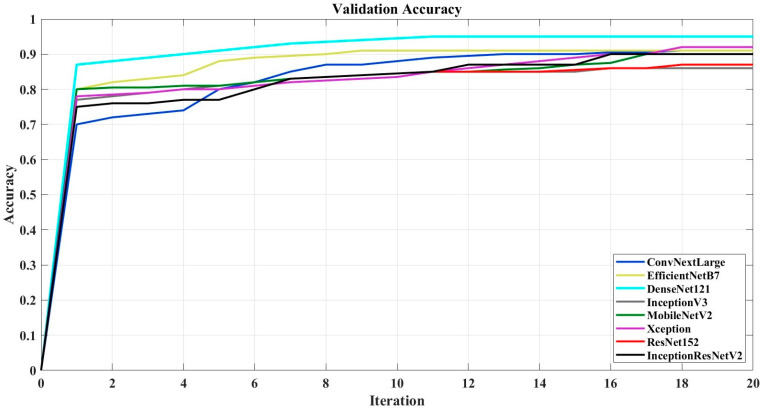
Validation accuracy.

In Figure 2, the validation accuracy graphs of the models are presented. The most successful models from the EfficientNet and ConvNeXt families have been included in the graph.

Upon examination of Figure 2, among the evaluated models, DenseNet121 demonstrates the highest validation accuracy, converging rapidly to near-perfect performance, thereby indicating its robustness and efficiency. EfficientNetB7 also achieves commendable performance, attaining a level of accuracy that is highly competitive, though marginally lower than that of DenseNet121. ConvNeXtLarge exhibits strong performance but does not quite attain the validation accuracy observed with DenseNet121 and EfficientNetB7. The remaining models—InceptionV3, MobileNetV2, Xception, ResNet152, and InceptionResNetV2—converge to relatively high accuracy values; however, they fall short when compared to the leading models. Notably, most of the models stabilize in terms of accuracy after approximately 10 to 15 iterations, suggesting that additional training beyond this point yields limited gains. In conclusion, DenseNet121 is distinguished by its superior accuracy, followed closely by EfficientNetB7, while the other architectures achieve comparable but slightly lower levels of performance.

**Figure 3:**
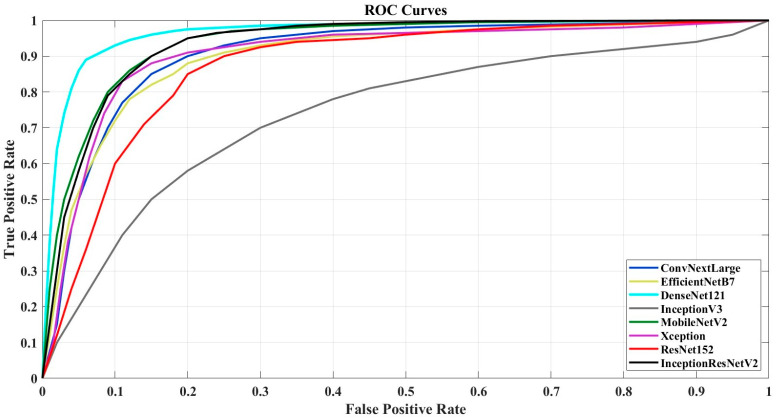
ROC curve.

The performance of these models was thoroughly evaluated to determine their accuracy in classification tasks. The results of these experiments are summarized in Table 3, which compares the predictive performance of each model based on various metrics, while Figure 3 displays the ROC (Receiver Operating Characteristic) curves. As in Figure 2, the most successful networks from the ConvNeXt and EfficientNet families are included in this graph.

When interpreting Table 3 in the context of classifying six different types of fungi, several insights can be drawn regarding the effectiveness of the various models. DenseNet121 stands out as the top performer, achieving an accuracy of 0.92 and an AUC of 0.95. This indicates that DenseNet121 is particularly effective at distinguishing between different fungi types while minimizing both false positives and false negatives. Its high precision and recall scores further suggest its ability to accurately identify the correct fungi species with a high degree of reliability. The ConvNeXt models also demonstrate strong performance, with ConvNeXtLarge reaching an accuracy of 0.89. The similar performance of ConvNeXtSmall and ConvNeXtBase, both achieving accuracy rates above 0.87, indicates that even the smaller variants of this model are capable of effectively classifying fungi. MobileNetV2 and Xception, each achieving 0.90 accuracy, further underscore their suitability for this classification task. These models strike a balance between high accuracy and computational efficiency, which is crucial when deploying classification systems in real-world or resource-constrained settings. In contrast, the EfficientNetB0 and B3 models, both scoring 0.82, appear to struggle with accurately classifying fungi images. Their lower precision and recall scores indicate a higher likelihood of misclassifications. However, EfficientNetB7 performs better, with an accuracy of 0.89, suggesting that scaling the EfficientNet architecture can lead to improved performance in fungi classification tasks. InceptionV3 demonstrates a more modest accuracy of 0.84, and its lower precision suggests it may face difficulties in reliably differentiating between various fungi types, making it less ideal for this particular task. ResNet152, with an accuracy of 0.87, proves capable of handling the complexities of fungi classification, making it a reliable option for researchers. However, swin transformers perform poorly, achieving an accuracy of only 0.64. This indicates that this architecture may not be suitable for fungi classification, potentially due to its limitations in capturing the subtle, nuanced features necessary for differentiating fungi types in RGB images.

Considering the ROC curves, DenseNet121 and MobileNetV2 demonstrate the best performance, with curves closest to the top-left corner, indicating a strong balance between high true-positive rates and low false-positive rates. InceptionResNetV2 also performs well, achieving nearly comparable results. EfficientNetB7, ConvNeXtLarge, Xception, and ResNet152 show decent though slightly less effective performance, with their ROC curves positioned further from the top-left corner. InceptionV3 has the weakest ROC curve, indicating a higher false-positive rate and a generally lower ability to distinguish between classes. Overall, DenseNet121 and MobileNetV2 are the top performers, while InceptionV3 is the least effective in this comparison.

**Figure 4:**
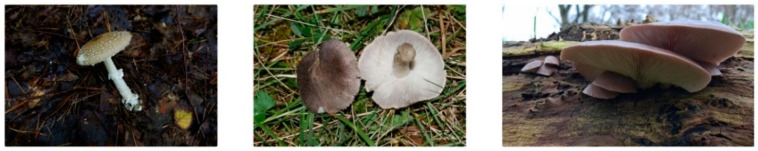
İmages without Grad-CAM visualization.

**Figure 5:**
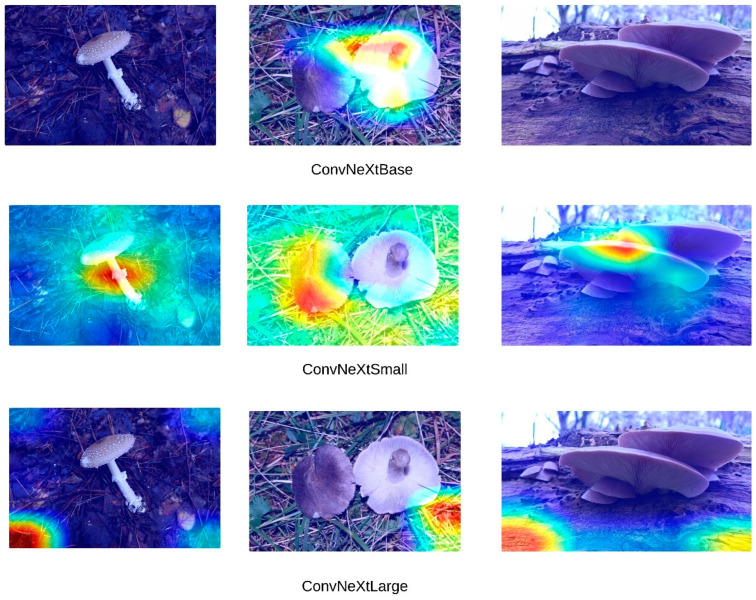
ConvNeXt Grad-CAM visualization.

**Figure 6:**
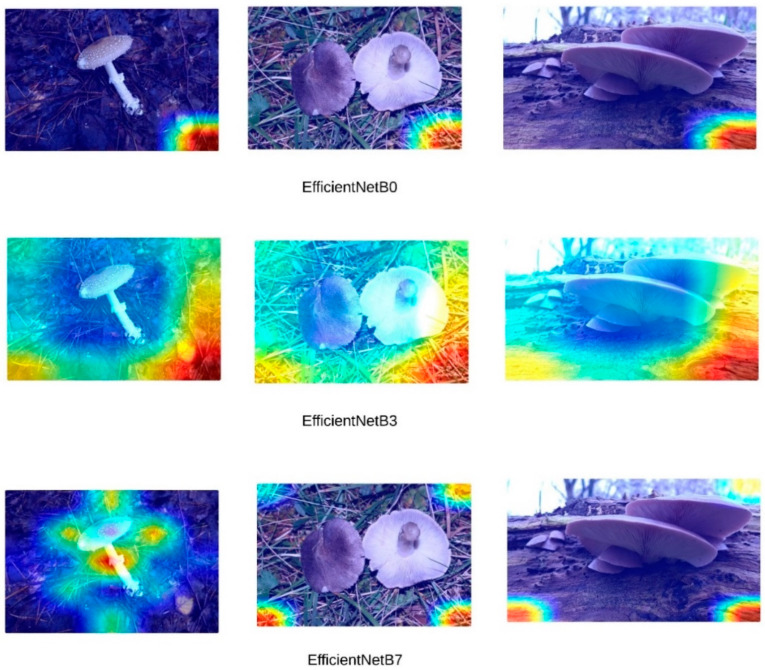
EfficientNet Grad-CAM visualization.

**Figure 7:**
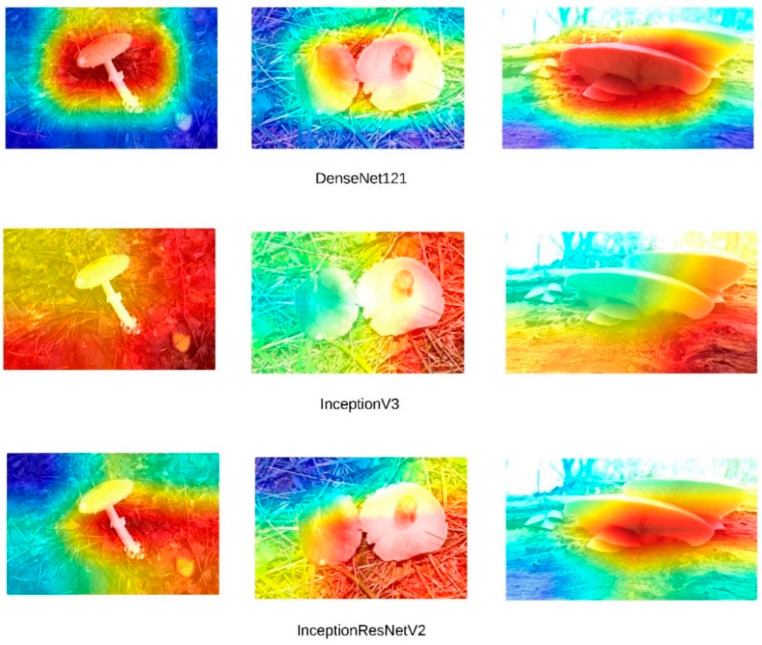
DenseNet121, InceptionV3, and InceptionResNetV2 Grad-CAM visualization.

**Figure 8:**
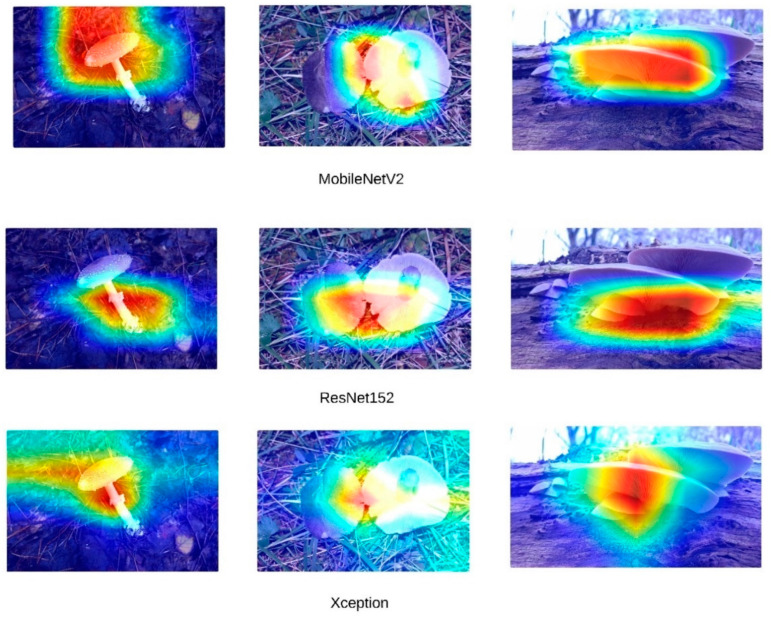
MobileNetV2, ResNet152, and Xception Grad-CAM visualization.

High accuracy rates do not necessarily indicate that models are making inferences based on the correct features. To address this, Grad-CAM (Gradient-weighted Class Activation Mapping) visualizations were generated for the three images presented in Figure 4. Figure 5 presents the Grad-CAM heatmaps for ConvNeXt, Figure 6 for EfficientNet, Figure 7 for DenseNet121, InceptionV3, and InceptionResNetV2, and Figure 8 for the MobileNetV2, ResNet152, and Xception models, allowing for an analysis of the specific regions from which the models derive their predictions. These visualizations provide critical insight into the areas of the images that influence the models’ classification decisions, facilitating the identification of potential misinterpretations or incorrect focal points.

Upon a detailed examination of the Grad-CAM images, it becomes evidently clear that DenseNet121 emerges as the most effective model for fungi classification. The Grad-CAM visualizations provide insight into the model’s ability to focus on the relevant regions of the images, highlighting its superior capability to correctly identify critical features associated with different fungi types. In contrast, the ConvNeXt models, which incorporate transformer-based architectures, exhibit less effective visual attention patterns. This suggests that transformer-based models, as reflected by ConvNeXt’s visualizations, are not well-suited for this specific task of fungi classification. The limited efficacy of transformers in this domain is further corroborated by the notably poor performance of the swin transformer model, which struggles to capture the distinguishing features of the fungi images. In comparison, MobileNetV2 demonstrates significant efficacy, ranking just behind DenseNet121. The Grad-CAM visualizations for MobileNetV2 suggest that it successfully focuses on important image regions, supporting its relatively strong performance metrics. This indicates that while MobileNetV2 may not reach the same level of accuracy as DenseNet121, it remains a highly competent model for fungi classification, especially in scenarios where computational efficiency is also a priority. Overall, the Grad-CAM analyses provide valuable insight into how these models perform, reinforcing the idea that models like DenseNet121 and MobileNetV2 are more suitable for this classification task than transformer-based architectures such as swin transformer. Swin transformers, like other transformer-based architectures, are designed to capture long-range dependencies by processing images in non-local patches, which works well for tasks with large-scale or global features. However, fungi images often consist of fine-grained textures and intricate local patterns that require precise, detailed feature extraction. CNNs, with their convolutional filters, are inherently better suited for such tasks, as they focus on local features, allowing them to more effectively capture the subtle structural details found in fungi. Additionally, transformer models typically perform optimally with large datasets, where their ability to model long-range dependencies can be fully exploited. Given that the dataset used in this study may not have been large or diverse enough, it is possible that swin transformers struggled to generalize, leading to overfitting or suboptimal performance.

To elucidate the robustness of the DenseNet121 and MobileNetV2 networks, which demonstrated the highest performance metrics, their effectiveness in classifying individual mushroom species and their performance on images contaminated with Gaussian noise were also evaluated.

**Figure 9:**
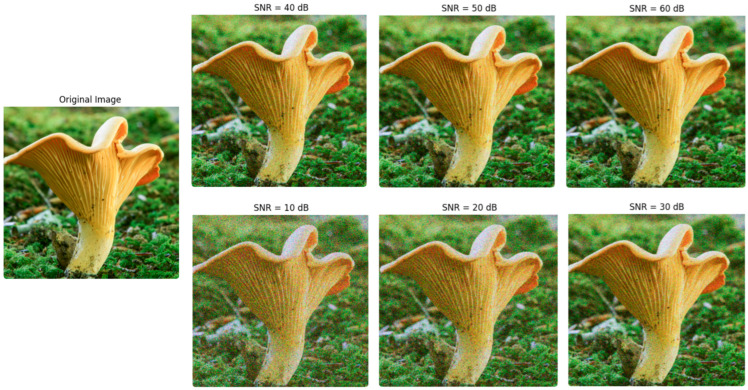
Different levels of Gaussian white noise [40].

Gaussian noise was introduced to the images across a Signal-to-Noise Ratio (SNR) range of 10 to 60 (10, 20, 30, 40, 50, 60) [40] to simulate various levels of degradation and evaluate model robustness under differing noise intensities. The detailed assessment of model performance focused primarily on experiments conducted at the highest noise levels within this range, as these levels present a more rigorous and challenging test of the models’ capacity to maintain accuracy in adverse conditions. Figure 9 presents images with noise applied at different levels.

The metrics in Table 4 indicate that the models are highly proficient in distinguishing between different fungal species, exhibiting minimal performance variance across categories. This consistency demonstrates the model’s suitability for applications requiring accurate and reliable detection and the classification of diverse fungal species.

Since deep networks are inherently capable of performing well on noisy images, noisy images did not need to be included during the training phase. Incorporating noise into the training data could obscure the networks’ true robustness by enabling them to learn specific noise patterns, rather than relying on their intrinsic capacity to handle noisy inputs. This approach allows for a more objective evaluation of the models’ resilience to noise, as any performance on noisy images in the testing phase reflects the models’ innate robustness rather than adaptive learning to noise during training. Evaluation across individual SNR values, ranging from 20 to 60, as well as for all noise levels combined, consistently yielded performance metrics of 0.89 or higher. This consistency across varying noise intensities indicates a high degree of stability in model performance, demonstrating that even in the presence of significant noise interference, the models retained strong accuracy and predictive power.

**Figure 10:**
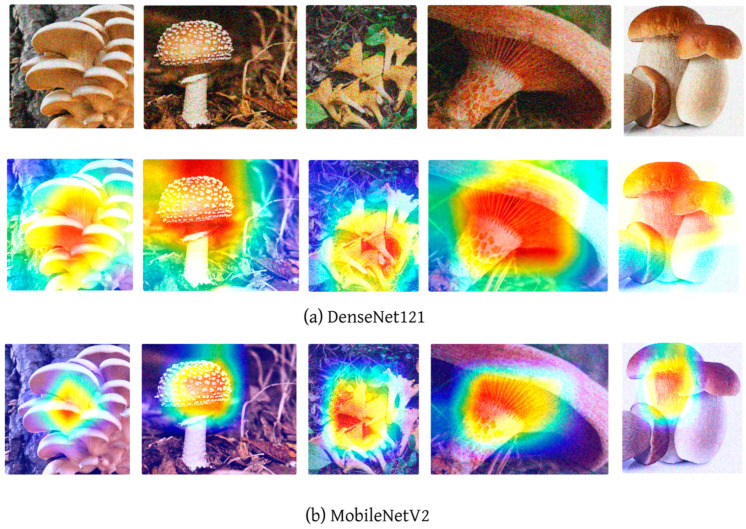
DenseNet121 and MobileNetV2 Grad-CAM visualization on SNR-10 noisy images.

The performance of DenseNet121 on SNR 10 noisy images, with an accuracy of 0.90, a precision of 0.89, a recall of 0.89, an F1-score of 0.90, and an AUC of 0.91, indicates that the model remains robust and effective even under challenging conditions. The high accuracy and balanced precision and recall demonstrate that DenseNet121 maintains a strong ability to correctly classify noisy inputs while minimizing false positives and false negatives. The F1-score of 0.90 reinforces the model’s capacity to handle noisy data effectively, as it shows a good balance between precision and recall. Additionally, the AUC of 0.91 reflects a high degree of separability between classes, indicating that the model can still distinguish between different categories despite the presence of noise. Overall, these results suggest that DenseNet121 is resilient to Gaussian noise and retains a high level of performance, making it suitable for applications where data may not always be clean or noise-free. Similarly, the performance metrics of MobileNetV2 on SNR-10 noisy images indicate a robust classification ability, with an accuracy of 0.87, reflecting that the model correctly identified 87% of instances in the dataset despite the noise. The F1-score, also at 0.87, demonstrates a well-balanced performance, effectively managing both precision and recall, which is crucial for applications where false positives and false negatives are significant. With a precision value of 0.90, the model indicates a high likelihood of correctness when predicting positive classes, thereby minimizing erroneous classifications. Meanwhile, a recall of 0.87 signifies that the model successfully detects 87% of actual positive instances, underscoring its effectiveness in identifying the target class. Finally, an AUC of 0.90 highlights MobileNetV2’s strong discriminatory power, illustrating its capability to distinguish between positive and negative instances across different classification thresholds. Collectively, these metrics suggest that MobileNetV2 performs reliably in classification tasks. Figure 10 shows the Grad-CAM colorization of DenseNet121 and MobileNetV2 on SNR-10 noisy images.

In summary, the results indicate that models DenseNet121 and MobileNetV2 are well-equipped to tackle the challenges of classifying RGB images of fungi, while models such as ML and swin transformers may require further optimization to be viable for this task. Future work could explore whether increasing the dataset size or employing hybrid architectures might help improve the performance of transformer-based models for fungi classification. Furthermore, applying image enhancement [41] techniques to low-quality fungi images could be explored to assess their potential impact on enhancing model performance.

## 4. Discussion

The results of this study demonstrate the effectiveness of deep learning models in the classification of mushrooms, with findings that align well with the existing literature on similar applications. DenseNet121, EfficientNet, and MobileNet emerged as the key models capable of handling the complexity and heterogeneity of mushroom image data, with DenseNet121 consistently proving the most effective. Specifically, DenseNet121 achieved high reliability across multiple studies, with reported accuracy rates reaching 98% [42,43,44]. This consistency highlights its strong capacity to capture essential features for accurate fungi classification. The observed performance differences between models, particularly during the training phases, underscore the importance of selecting appropriate architectures for tasks involving complex image data.

Convolutional neural networks (CNNs) have shown significant potential in plant disease detection and similar image classification tasks, and this study affirms their utility for fungal species identification [45,46,47]. For instance, CNN-based models have previously demonstrated high classification accuracy in detecting diseases such as rice blight, achieving rates up to 97.7% [45]. Such success translates well to the context of fungal infections, suggesting CNN models are powerful tools for agricultural disease management and ecological studies involving fungi [45,46,47].

A critical factor affecting the performance of deep learning models is the size and diversity of the training dataset. As widely evidenced in previous research, larger and more varied datasets contribute substantially to the accuracy and generalizability of deep learning models [48,49,50]. In this study, expanding the dataset through augmentation techniques improved classification accuracy and enhanced the models’ robustness. These methods have successfully minimized misclassification rates and facilitated the more efficient detection of fruit body emergence, particularly when the models are trained on constrained datasets. By enhancing accuracy, these approaches ensure that the detection process remains reliable and effective even with limited data [51,52].

In addition to deep learning, methods such as transfer learning and ensemble modeling provide significant advantages in fungal classification and detection. By leveraging pre-trained models on large datasets, transfer learning enables accurate classification even when training data are scarce. It has been noted that models using transfer learning often outperform those trained from scratch by a margin of 10–15%, consistent with the results obtained here [53,54,55]. Ensemble modeling approaches also showed significant promise; combining multiple models (e.g., the DEX ensemble comprising DenseNet121, EfficientNetB7, and Xception) reduced classification errors and achieved accuracy up to 98%, enhancing the precision of ecological monitoring [53,54,55,56,57,58,59].

DenseNet121 emerged as the most effective model in this study, achieving the highest accuracy of 0.92 and an AUC of 0.95, indicating its superior ability to distinguish between different types of fungi with minimal false positives and negatives [43,44,45,46,47,48,49,50,51,52,53,54,55,56,57,58]. Its strong precision and recall metrics further reinforce its utility in accurately identifying fungi species, making it highly reliable for classification tasks. Similarly, ConvNeXtLarge also demonstrated solid performance, although its overall accuracy of 0.89 fell slightly behind DenseNet121 [59,60,61,62]. Visual analyses using Grad-CAM, however, indicated that ConvNeXt models exhibited less effective attention on crucial features, particularly compared to DenseNet121. This highlights a limitation of current transformer-based architectures, as they may lack the sensitivity required for detailed feature extraction in fungal image classification [63,64].

The performance of transformer-based models varied significantly. While ConvNeXtSmall and ConvNeXtBase showed moderate success, the swin transformer performed poorly, achieving an accuracy of only 0.64 [65,66,67]. These findings suggest that, in their current form, transformer architectures are not as suitable for this task compared to CNN-based models. Further optimizations or architectural modifications might be necessary to enhance their applicability in fungal classification [67,68,69,70,71,72,73,74,75].

Among the CNN models tested, MobileNetV2 demonstrated high performance with an accuracy of 0.90. Its computational efficiency makes it an attractive option for practical use, particularly in resource-constrained environments [75,76,77,78]. The Grad-CAM visualizations for MobileNetV2 indicated its capability to focus on relevant regions in the images, reinforcing its efficacy for the task. Xception also performed well, with an accuracy of 0.90, though other models like InceptionV3, with an accuracy of only 0.84, struggled to match this level of performance [79,80,81]. The EfficientNet family showed mixed results; while EfficientNetB7 achieved an accuracy of 0.89, models like EfficientNetB0 and EfficientNetB3 performed less effectively, with an accuracy of around 0.82 [82,83,84,85].

ResNet152, achieving an accuracy of 0.87, demonstrated its ability to handle the complexities of the dataset, although it was outperformed by more recent models such as DenseNet121 and MobileNetV2 [86]. This suggests that newer architectures with more advanced feature extraction capabilities provide better solutions for complex biological imagery tasks [87].

In summary, this study highlights the effectiveness of DenseNet121 and MobileNetV2 for fungi classification, as evidenced by their quantitative performance and visual attention analyses using Grad-CAM [88]. DenseNet121’s superior ability to capture crucial image features, coupled with MobileNetV2’s computational efficiency, makes them highly suitable for practical deployment. However, the limited effectiveness of traditional ML techniques and transformer-based architectures, particularly the swin transformer, indicates the need for further refinement or optimization in this domain.

## 5. Conclusions

This study investigated the application of deep learning models for classifying six different macrofungi species using RGB images. The comparative analysis demonstrated that DenseNet121 was the most effective model, achieving the highest accuracy (92%) and AUC (95%) scores. MobileNetV2 also proved to be a strong contender, offering a good balance of performance and computational efficiency. These results indicate that DenseNet121 and MobileNetV2 are well-suited for practical fungi classification tasks, particularly in environments with limited computational resources.

Transformer-based models, however, particularly the swin transformer, did not perform as well as CNN-based models in this study. Their relatively poor performance underscores transformers’ challenges in distinguishing between complex fungal features in RGB images. This suggests they may require further optimization or modification for practical use in this domain.

For future research, expanding and diversifying the dataset is recommended to improve the generalizability of the models. The use of ensemble learning techniques, combining the strengths of different models, could further enhance classification accuracy. Moreover, integrating additional data types, such as biochemical properties, scanning electron microscopy (SEM), and light microscopy images, may offer deeper insights into fungal features, improving model performance. Incorporating RNA/DNA sequencing data would also allow for genetic-level identification, providing a more comprehensive understanding beyond just morphological classification. These advancements could lead to more precise and reliable methods for ecological monitoring and agricultural management of fungi.

## Figures and Tables

**Table 1:** Type names of datasets taken from open sources, source name, percentage share of the taken dataset in the entire used dataset, and the technical specifications of the sources.

Mushroom Species Name	Source	Percentage of Photographs Taken from Source	Photo Type	Resolution
*Amanita pantherina*	Global Core Biodata Resource	<25%	JPG	300 dpi
*Boletus edulis*	Global Core Biodata Resource	<20%	JPG	300 dpi
*Cantharellus cibarius*	Global Core Biodata Resource	<25%	JPG	300 dpi
*Lactarius deliciosus*	Global Core Biodata Resource	<25%	JPG	300 dpi
*Pleurotus ostreatus*	Global Core Biodata Resource	<25%	JPG	300 dpi
*Tricholoma terreum*	Global Core Biodata Resource	<25%	JPG	300 dpi

**Table 2:** Experimental results for ML.

	Accuracy	Precision	Recall	F1-Score	AUC
Logistic regression	0.60	0.60	0.60	0.58	0.70
**SVM**	**0.60**	**0.60**	**0.60**	**0.56**	**0.71**
k-NN	0.45	0.42	0.45	0.40	0.50
Decision tree	0.48	0.47	0.48	0.48	0.53
Random forest	0.57	0.57	0.57	0.54	0.60

**Table 3:** Experimental results for DL.

Models	Accuracy	Precision	Recall	F1-Score	AUC
ConvNeXtBase	0.87	0.87	0.87	0.87	0.87
ConvNeXtSmall	0.88	0.87	0.87	0.87	0.87
ConvNeXtLarge	0.89	0.89	0.90	0.90	0.90
EfficientNetB0	0.82	0.78	0.78	0.79	0.80
EfficientNetB3	0.82	0.78	0.78	0.78	0.80
EfficientNetB7	0.89	0.90	0.88	0.89	0.90
**DenseNet121**	**0.92**	**0.94**	**0.90**	**0.92**	**0.95**
InceptionV3	0.84	0.73	0.73	0.73	0.80
MobileNetV2	0.90	0.93	0.90	0.90	0.92
Xception	0.90	0.92	0.90	0.90	0.90
ResNet152	0.87	0.92	0.82	0.87	0.90
InceptionResNetV2	0.90	0.92	0.87	0.90	0.90
Swin transformers	0.64	0.75	0.60	0.63	0.60

**Table 4:** Results of DenseNet121 and MobileNetV2 on fungal species.

	Model	Accuracy	Precision	Recall	F1-Score
*Amanita pantherina*	DenseNet121	0.92	0.94	0.9	0.92
*Boletus edulis*		0.918	0.94	0.9	0.92
*Cantharellus cibarius*		0.92	0.94	0.9	0.918
*Lactarius deliciosus*		0.92	0.94	0.9	0.92
*Pleurotus ostreatus*		0.92	0.94	0.9	0.918
*Tricholoma terreum*		0.92	0.94	0.9	0.92
*Amanita pantherina*	MobileNetV2	0.9	0.93	0.9	0.9
*Boletus edulis*		0.899	0.92	0.899	0.899
*Cantharellus cibarius*		0.899	0.928	0.899	0.899
*Lactarius deliciosus*		0.9	0.93	0.9	0.9
*Pleurotus ostreatus*		0.9	0.93	0.9	0.9
*Tricholoma terreum*		0.9	0.93	0.9	0.9

## Data Availability

The raw data supporting the conclusions of this article will be made available by the authors on request.
